# Multi-informant Examination of Cognitive Disengagement Syndrome in Relation to Sleep and Circadian Preference in Early Adolescents

**DOI:** 10.1007/s10578-026-01990-z

**Published:** 2026-04-07

**Authors:** Melissa C. Miller, James L. Peugh, Jeffery N. Epstein, Leanne Tamm, Stephen P. Becker

**Affiliations:** 1 Division of Behavioral Medicine and Clinical Psychology, Cincinnati Children’s Hospital Medical Center, 3333 Burnet Avenue, MLC 10006, Cincinnati, OH 45229-3039, USA; 2 Department of Pediatrics, University of Cincinnati College of Medicine, Cincinnati, OH, USA

**Keywords:** Adolescence, cognitive disengagement syndrome, Sluggish cognitive tempo, Sleep, Circadian

## Abstract

Considerable research has documented an association between attention-deficit/hyperactivity disorder (ADHD) and poor sleep functioning, yet few studies have examined the potential role of frequently co-occurring cognitive disengagement syndrome (CDS). Further, extant studies have used non-optimal measures of CDS and/or sleep, small sample sizes, or rarely focus on adolescence when sleep problems often emerge and worsen, and few have controlled for depressive symptoms which are strongly associated with both CDS and poorer sleep functioning. Accordingly, we used a multi-informant design to examine CDS, ADHD, and depressive symptoms in relation to sleep disturbance, daytime sleep-related impairment, and circadian preference in a sample of 341 early adolescents (ages 10–12). Adolescents, caregivers, and teachers completed measures of CDS, ADHD, and depression. Adolescents and caregivers completed the patient-reported outcome measurement information system (PROMIS) sleep disturbance and sleep-related impairment scales; adolescents also completed a measure of circadian preference. Multivariate regression analyses examined CDS, ADHD, and depressive symptoms in relation to sleep functioning. Across models with different informants, CDS symptoms were more consistently associated than ADHD dimensions with greater sleep-related impairment and sleep disturbance. In contrast, only self-reported ADHD inattentive symptoms were independently associated with greater eveningness preference. This study provides further evidence of the unique association between CDS and sleep disturbance and sleep-related impairment, raising the possibility that the established association between ADHD and sleep disturbance may be in part due to or exacerbated by co-occurring CDS. Additional longitudinal research is needed to examine the directionality of effects and impact of sleep interventions on CDS.

Cognitive disengagement syndrome (CDS), previously referred to as sluggish cognitive tempo, is characterized by a set of developmentally inappropriate cognitive and motor symptoms such as excessive daydreaming, mental confusion, and hypoactivity [15]. Originally posited to be a way to identify a “pure” attention deficit/hyperactivity disorder (ADHD) inattentive group, accumulating research over the last two decades has evidenced that CDS symptoms are distinct from, yet strongly associated with, ADHD inattentive symptoms [12, 15]. The distinct nature of CDS is evidenced by studies finding that CDS is more strongly associated with internalizing compared to externalizing symptoms when controlling for ADHD, with a particularly strong association with depression [5]. Importantly, studies have shown that CDS is also distinct from internalizing symptoms including anxiety and depression [43, 51, 65, 68]. As evidence of the distinct nature has accumulated, focus has turned to identifying functional impairment associated with CDS, especially among domains of impairment that have been linked with ADHD, with a growing body of research finding CDS to be independently associated with numerous areas of impairment, above and beyond ADHD symptoms, including academic, social, and cognitive functioning deficits [15, 38].

It is well-established that ADHD is associated with sleep and sleep-related difficulties [3, 17, 47], as well as later circadian phase and evening diurnal preference (e.g., preference for greater activity during the evening or night hours) [11, 27]. Different patterns of sleep difficulties have been identified based on ADHD symptoms and subtypes [17]. Previous research suggests that hyperactivity-impulsivity is associated with greater nighttime sleep difficulties, including short sleep duration [34, 58], whereas inattention is associated with greater daytime sleep difficulties, including inadvertent daytime napping, hypersomnia [24], and daytime sleepiness [42, 50]. Given the association between ADHD inattention and daytime sleepiness, the association between ADHD inattention and CDS, as well as the hypoactivity, excessive drowsiness, and lethargy that characterize CDS, the association between CDS and sleep functioning has recently emerged as an area of growing research focus [38].

A small but growing body of research with both child and adult samples has found associations between CDS symptoms and multiple domains of sleep functioning, including daytime sleepiness [10, 14], poor sleep quality [14], sleep problem severity [10], greater evening preference [49], and overall sleep difficulties [18, 53, 61]. Mayes et al. [52] found sleeping more than normal was a strong correlate of higher CDS symptoms in a population-based sample of children ages 6–12 years. Importantly, one study found that sleep problems longitudinally predicted CDS symptoms, but CDS symptoms did not predict future sleep problems, providing some insight on possible directionality between sleep and CDS [53]. Among studies that have examined differential associations between CDS and ADHD subtypes or symptoms, stronger associations between CDS and sleep difficulties and poorer sleep quality have been observed [14, 61]. However, findings from many of these studies are limited in their generalizability due to their methodology, including reliance on exclusively-ADHD clinical samples [10, 49, 61] or use of suboptimal measures of CDS (e.g., ad hoc measure that does not include the full range of CDS items, or measures that include items that are not core symptoms of the CDS construct) [10, 53, 61]. Other studies relied exclusively on caregiver-report [10, 18, 53]. As caregivers begin to have less of a role in managing their child’s sleep routine during adolescence, obtaining ratings from other informants may be critical to understanding one’s functioning. For example, teachers are valuable reporters of adolescents’ day-to-day functioning [66, 69] and teacher-report of symptoms may be especially illuminating in regard to negative impacts related to poor or insufficient sleep (e.g., daytime sleepiness) [19]. However, no studies have examined teacher-reported CDS symptoms in relation to adolescent’s sleep and sleep-related impairment.

Examining CDS and sleep in adolescents is especially important given biological changes (e.g., slowed sleep pressure; circadian phase delays) and psychosocial factors (e.g., increased autonomy surrounding bedtime; academic pressure) that occur during adolescent development [22, 28]. Carskadon, [22] suggested these changes result in a “perfect storm” of inadequate sleep and subsequent consequences for adolescents. When considering examining sleep in adolescents, it is potentially critical to include adolescent self-report measures because caregivers are less aware of the sleep patterns and problems in their adolescent children [54, 59]. Smith and colleagues [65] found CDS to be significantly associated with self-reported daytime sleepiness, but the sample consisted entirely of youth with ADHD (ages 10–15) and the study used a measure of CDS that includes items that have been found to load with ADHD inattention rather than on a unique CDS factor [4], making it important to replicate and extend these findings.

More recently, Fredrick and colleagues [33], using a measure of self-reported CDS with strong psychometric properties and both caregiver and self-report informants for sleep functioning, reported significant associations between self-reported CDS and multiple areas of sleep functioning among adolescents (ages 12–14), including self-reported sleep/wake problems, caregiver-reported difficulties initiating/maintaining sleep, and both self- and caregiver-reported daytime sleepiness, above and beyond demographics and ADHD status. In addition, self-reported CDS symptoms were associated with sleep functioning as assessed by daily diary ratings (i.e., later bedtime, longer sleep onset latency, greater night wakings, longer duration of wake after sleep onset, shorter sleep duration, greater difficulty waking in the morning) and actigraphy (i.e., later sleep onset time, fewer minutes in bed). In a study of 62 adolescents (ages 13–17) with and without ADHD, Lunsford-Avery et al. [48] found several polysomnography-assessed sleep physiology indices (e.g., nocturnal awakenings, reduced sleep efficiency, greater proportion of the night spent in light sleep) were associated with greater parent-reported CDS symptoms, with findings largely robust to control for ADHD symptom severity. However, neither Fredrick et al. nor Lunsford-Avery et al. included depressive symptoms in their analyses, potentially clouding the association between CDS and sleep domains given the strong association depression has with both CDS [62] and sleep [46] in adolescence. In addition to significant changes in sleep during adolescence, there are also related but distinct changes in circadian function [28]. Two studies reported CDS among adolescents with and without ADHD is associated with later eveningness circadian preference (e.g., preference for activity in evening or night hours) [33]. Despite circadian shifts toward later bedtimes in adolescence, school start times largely remain the same, which can lead to shorter sleep durations and irregular sleep patterns [28, 35, 63]. Importantly, one study found that having less than optimal sleep (e.g., restricted to 6.5 h) causally contributes to increases in both caregiver- and adolescent-reported CDS symptoms in adolescents (ages 14–17) with ADHD [8].

Given the emerging literature suggesting an important link between CDS and sleep, the current study aimed to extend existing findings by using a multi-informant approach with well-validated measures of CDS symptoms and sleep to examine CDS, ADHD, and depressive symptoms in relation to sleep disturbance, daytime sleep-related impairment, and circadian preference in a community-based sample of early adolescents. Specifically, we used adolescent, caregiver, and teacher ratings of CDS, ADHD, and depressive symptoms, as well as adolescent and caregiver ratings of sleep disturbance and sleep-related impairment.

## Methods

### Participants

Participants were 341 early adolescents (ages 10–12 years; *M*±*SD*_age_ = 10.90 ± 0.80; 52.7% female; 38.1% non-White; 9.5% Hispanic/Latine). Approximately half (48.7%) of the sample met diagnostic criteria for ADHD based on the Kiddie Schedule for Affective Disorders and Schizophrenia for School-Age Children (K-SADS [39]) conducted with the adolescent’s caregiver (primarily biological mothers). Participants who provided data for the current study were recruited as part of a larger study investigating emotions and behaviors associated with CDS symptoms. All data evaluated in the present study were collected at baseline visits conducted between 2021 and 2023. Characteristics of the sample are summarized in Table 1.

### Procedures

All procedures were approved by the Cincinnati Children’s Hospital Medical Center Institutional Review Board. Participants were recruited from a variety of sources, including media advertisements, community flyers, e-mail distribution within a Midwestern children’s hospital, and letters to school and pediatrician partners. This study purposefully used a variety of recruitment materials to ensure a full range of CDS and attention symptomatology was represented for dimensional-focused analyses (e.g., some materials included descriptors of CDS and ADHD symptoms, some materials omitted descriptors of attentional concerns). There was no requirement for elevations in CDS or any other psychopathology. Interested caregivers completed a brief REDCap survey that included initial inclusion criteria (e.g., child’s age); those that met criteria were scheduled for an in-person research visit. After caregivers and adolescents provided informed consent and assent, respectively, study measures were collected. Caregivers provided contact information for the adolescent’s teacher to collect teacher-report measures.

Inclusion criteria included being between 10 and 12 years of age, a receptive vocabulary (an index of intellectual ability) standard score ≥ 80 on the Peabody Picture Vocabulary Test, 5th Edition [29], willingness to withhold ADHD stimulant medications the day of their research visit, and sufficient English language ability to complete measures. Exclusion criteria included adolescent diagnoses of autism spectrum disorder, bipolar disorder, psychosis, either reported as historic diagnoses by the caregiver or based on the KSADS conducted with the caregiver. In addition, exclusion criteria included significant visual, hearing or speech impairment precluding their ability to complete measures.

### Measures

#### Demographic Characteristics and Medication Use

Caregivers completed a demographic form to gather the information reported in Table 1. Medication use and psychosocial treatment was assessed with an adapted Services Use in Children and Adolescents-Parent Interview (SCA-PI [30]).

#### Clinical Interview

##### Kiddie Schedule for Affective Disorders and Schizophrenia for School-Age Children (K-SADS)

The K-SADS [39] is a semi-structured diagnostic interview based on the DSM-5 with demonstrated reliability and validity [37]. In the present study, K-SADS was used to assess ADHD, oppositional defiant disorder (ODD), and conduct disorder (CD), anxiety disorders, and depressive disorders in caregiver interviews, whereas interviews conducted with the adolescent were used to assess anxiety and depressive disorders.

#### CDS, ADHD, and Depressive Symptoms

##### Child and Adolescent Behavior Inventory (CABI)

Caregivers and teachers completed the 15-item CDS, 6-item depression, 9-item ADHD-inattention (ADHD-IN), and 9-item ADHD-hyperactivity/impulsivity (ADHD-HI) scales from the CABI [20]. Each item is rated on a six-point scale for the past month (0 = *almost never [never or about once per month]* to 5 = *almost always [many times per day]*). Scores on the 15 CABI CDS items (e.g., “gets lost in own thoughts,” “easily confused,” “daydreams”, “low level of activity”) have demonstrated strong structural validity (including discriminant validity from ADHD and other psychopathology symptoms including depression, anxiety, and oppositionality), excellent reliability (internal consistency, test-retest, inter-rater), invariance (across a one-month interval, sex of rater, community/clinical samples), and independent correlates relative to ADHD symptoms (for a review, see Ref. [4]). Because of item overlap with measures of sleep functioning, two CDS items (i.e., “drowsy or sleepy during the day,” “easily tired or fatigued”) were omitted from analyses to reduce risk of conflation. Reliability (i.e., alpha) for the current sample of participants for the caregiver and (teacher) CABI CDS, depression, ADHD-IN, and ADHD-HI scores were 0.94 (0.96), 0.85 (0.88), 0.98 (0.96), and 0.93 (0.95), respectively. Mean CDS, depression, ADHD-IN, and ADHD-HI item scores were used in the analyses in the current study, with higher scores indicating higher frequency of symptoms.

##### Child Concentration Inventory, Second Edition (CCI-2)

Adolescent self-report of CDS was measured using the 15-item CCI-2 [2]. CCI-2 items parallel the items of the CABI CDS scale (e.g., “I get lost in my own thoughts,” “I feel confused”). Items are rated on a four-point scale (0 = *never* to 3 = *always*). As with the CABI, two CCI-2 items (i.e., “I feel sleepy or drowsy during the day,” “I get tired easily”) with overlap with sleep items were removed. Reliability (i.e., alpha) was 0.87 for CCI-2 scores in this study. A mean CCI-2 item score was used in the analyses with higher scores indicating a higher frequency of CDS symptoms.

##### Behavioral Assessment System for Children, Third Edition (BASC-3)

The BASC-3 Self-Report of Personality (SRP) [60] is a reliable and well-validated multidimensional measure to evaluate emotional and behavioral functioning in youth. For the current study, the Depression, Attention Problems, and Hyperactivity clinical scales from the BASC-3 SRP were used. Some items are true/false items and others are rated on a four-point scale (1 = *Never* to 4 = *Almost Always*). As in other studies examining ADHD symptom dimensions [1, 25], the BASC-3 SRP Attention Problems and Hyperactivity clinical scales were used to obtain self-report measures of ADHD-IN and ADHD-HI. Total raw scores were utilized in the current study, with higher scores indicating greater difficulties.

#### Sleep Functioning

##### PROMIS Sleep Disturbance and Sleep-Related Impairment Scales

Adolescents completed the 27-item Sleep Disturbance and 16-item Sleep-Related Impairment PROMIS scales [16, 32]. Caregivers completed the parent-proxy Sleep Disturbance (15 items) and Sleep-Related Impairment (13 items) scales. The PROMIS Sleep Disturbance Scale assesses perceptions of sleep quality, sleep depth, and restoration associated with sleep, including perceived difficulties and concerns with getting to sleep or staying asleep, as well as perceptions of the adequacy of and satisfaction with sleep. The PROMIS Sleep-Related Impairment Scale assesses perceptions of alertness, sleepiness, and tiredness during waking hours, as well as the perceived functional impairments during wakefulness associated with sleep problems or impaired alertness. Items for both scales are rated on a 5-point scale (1 = *never* to 5 = *always*), with higher scores indicating greater difficulties. The scales have demonstrated strong reliability and validity [6, 16, 32]. In this study, reliability (α) for the self-reported PROMIS Sleep Disturbance and Sleep-Related Impairment scales were 0.86 and 0.94, respectively. Reliability for caregiver-reported Sleep Disturbance and Sleep-Related Impairment scales were 0.95 and 0.94, respectively. Mean scale scores were used in the analyses with higher scores indicating a greater impairment.

##### Children’s Morningness-Eveningness Preferences (CMEP) Scale

The CMEP [23, 64] is an adolescent self-report measure of circadian preferences. The CMEP includes 10-items that reflect a range of preferences for different activities (e.g., “*Guess what? Your parents have decided to let you set your own bed time. What time would you pick?*”). The 10-item measure was adapted from the Morningness-Eveningness Questionnaire (MEQ [36]), a well-validated measure of circadian preferences in adults. Total CMEP scores were used in this study (α = 0.74), with lower scores indicating greater evening circadian preference and higher scores indicating greater morning preference.

### Analyses

Analyses were conducted using Mplus statistical software [56] using the maximum likelihood with robust standard estimator (MLR). Bivariate correlations were conducted to examine associations among demographic variables, psychopathology dimensions as measured by the CABI, BASC-SRP, and CCI-2, and sleep functioning as measured by PROMIS sleep scales and CMEP. Point-serial correlations were conducted when variables were dichotomous (i.e., medication use, sex, race). As a rule of thumb, a correlation of 0.10 is considered a small (weak) effect, 0.30 is considered a medium (moderate) effect, and 0.50 is considered a large (strong) effect [26]. Three separate multivariate multiple linear regression analyses were performed. All five sleep outcome variables (caregiver- and adolescent-rated sleep disturbance scores, caregiver- and self-rated sleep impairment scores, and self-rated circadian preference score) were included in each model. The three analyses differed only by whether caregivers, teachers, or adolescent self-report ratings of CDS, ADHD-IN, ADHD-HI, and depressive scores were included as predictors. All three models additionally included binary indicators of whether a participant was taking prescription medication (0 = no, 1 = yes), biological sex (0 = male, 1 = female), race (0 = person of color, 1 = White), and an ordinal indicator of annual income (1 = under $20k, 7 = over $120k) as control covariates. Pairwise missing data for all three multivariate analyses (range: 0.003–16.1%) was handled via maximum likelihood estimation to ensure the full sample was available for analysis. Alpha level for significance tests was set at *p* < .05 for all analyses.

## Results

### Bivariate Correlations

Most psychopathology dimensions (CDS, ADHD-IN, ADHD-HI, depression) were significantly positively correlated with each other (see Table 2, which also includes descriptive statistics of primary study variables; correlations involving covariates are provided in Table S1). Correlations between the two ADHD sub-dimensions ranged from small to large (*r*s=0.23–0.74, all *p*s<0.01) with stronger associations when the informant was the same (*r*s=0.60–0.74). Correlations between ADHD-IN and CDS emerged in a similar manner (*r*s=0.22–0.71, all *p*s<0.01; within-informant *r*s=0.69–0.71). Associations between ADHD-HI and CDS were lower and some were nonsignificant (*r*s=0.100.58). Correlations between depression and CDS ranged from small to moderate (*r*s=0.14–0.45, all *p*s<0.05) with stronger associations when the informant was the same (*r*s=0.41–0.45). Similarly, correlations between depression and ADHD-IN ranged from small to large (*r*s=0.12–0.48, all *p*s<0.05) with stronger within-informant associations (*r*s=0.34–0.48). The association between depression and ADHD-HI was strongest when both were reported by the adolescent (*r*=.49, *p*<.001). All other associations between depression and ADHD-HI were lower and some were nonsignificant (*r*s=0.03–0.25). Teacher-reported ADHD-HI was not significantly associated with caregiver-reported CDS or depression. Similarly, caregiver-reported ADHD-HI was not significantly associated with teacher-reported depression.

Regarding sleep variables, caregiver- and self-reported CDS, ADHD-IN, ADHD-HI, and depression were all significantly positively associated with both caregiver- and self-reported sleep disturbance (caregiver-reported *r*s=0.19–0.35, all *p*s<0.01; self-reported *r*s=0.17–0.52, all *p*s<0.01) and sleep impairment (caregiver-reported *r*s=0.13–0.41; self-reported *r*s=0.18–0.51, all *p*s<0.01). Teacher-reported CDS, ADHD-IN, and depression were significantly associated with self-reported sleep disturbance (*r*s=0.15–0.21, *p*s<0.05) and both self- and caregiver-reported sleep impairment (*r*s=0.15–0.26). Teacher-reported depression was significantly associated with caregiver-reported sleep disturbance (*r*=.12, *p*<.05) and teacher-reported ADHD-HI was significantly associated with self-reported sleep disturbance (*r*=.23, *p*<.01). Finally, self-reported circadian preference was significantly negatively associated with all self-reported psychopathology dimensions (*r*s=−0.18–0.32, *p*s<0.01), teacher-reported ADHD-HI (*r*=−.13, *p*<.05), and caregiver-reported depression (*r*=−.13, *p*<.05).

### Multivariate Regression Analyses

Multivariate analysis results are presented in Table 3. Results from caregiver independent variables showed several significant results. Specifically, increases in caregiver-reported CDS scores significantly predicted increases in caregiver- (*β* = 0.19, *p*=.009) and adolescent-reported sleep impairment (*β* = 0.18, *p*=.020) scores. Further results showed higher caregiver-reported ADHD-HI significantly predicted caregiver-reported sleep disturbance (*β* = 0.20, *p*=.005). Finally, caregiver-reported depressive symptoms were significantly associated with caregiver-reported sleep disturbance (*β* = 0.20, *p*<.001) and sleep-related impairment (*β* = 0.28, *p*<.001), as well as adolescent-reported sleep-related impairment (*β* = 0.17, *p*=.002) and circadian preference (*β*=−0.13, *p*=.038).

Multivariate analysis results from teacher independent variables showed two significant results: teacher-reported CDS scores significantly predicted increases in caregiver-reported (*β* = 0.19, *p*=.033) and adolescent-reported (*β* = 0.19, *p*=.029) sleep impairment. Teacher-reported depressive symptoms were significantly associated with caregiver-reported (*β* = 0.18, *p*=.006) and adolescent-reported (*β* = 0.16, *p*=.012) sleep impairment, and teacher-reported ADHD-HI was significantly associated with adolescent-reported sleep disturbance (*β* = 0.17, *p*=.024).

Multivariate analysis results from adolescent independent variables showed multiple significant results: (1) increases in adolescent-reported CDS scores significantly predicted increases in caregiver-reported sleep disturbance (*β* = 0.17, *p*=.021); (2) increases in adolescent-reported ADHD-IN (*β* = 0.22, *p*=.011) and decreases in adolescent-reported ADHD-HI scores (*β*=−0.22, *p*=.006) significantly predicted increases caregiver-reported sleep impairment; (3) increases in adolescent-reported CDS (*β* = 0.17, *p*=.005) and depression scores (*β* = 0.36, *p*<.001) predicted significant increases in adolescent-reported sleep disturbance; (4) increases in adolescent-reported CDS (*β* = 0.19, *p*=.002) and depression scores (*β* = 0.35, *p*<.001) significantly predicted increases in adolescent-reported sleep impairment, and (5) decreases in adolescent-reported ADHD-IN scores predicted significantly greater (*β*=−0.28, *p*=.001) adolescent-reported circadian eveningness preference.

## Discussion

Using a multi-informant design and including commonly co-occurring psychopathology symptoms (i.e., ADHD, depression), the current study extends the current understanding of the unique association CDS and sleep in adolescents, an important developmental period characterized by substantial changes in sleep and circadian functioning [22, 28]. Consistent with previous research, CDS symptoms were uniquely associated with sleep disturbance and sleep-related impairment, above and beyond symptoms of ADHD and depression, though these significant associations differed based on informant of CDS and sleep domains. Notably, these associations were observed utilizing a measure of CDS that omitted items that capture daytime sleepiness (e.g., “I feel sleepy or drowsy during the day,” “I get tired easily.”), ensuring that results were not attributable to the overlap between those CDS symptoms and sleep difficulties. Conversely, CDS symptoms were not significantly associated with circadian preference, regardless of informant of CDS symptoms.

Self-reported CDS symptoms, but not caregiver- or teacher-reported CDS symptoms, were associated with caregiver- and self-reported sleep disturbance (e.g., difficulties falling or staying asleep). Notably, ADHD and depressive symptoms were not significantly associated with caregiver-reported sleep disturbance. The lack of association between caregiver-reported CDS symptoms and sleep disturbance may be due to caregivers being less aware of adolescents’ sleep patterns and difficulties [31]. Further, the CDS symptoms that teachers observe in school are in a different context from where adolescent sleep occurs, and so the lack of an association based on teacher-reported CDS is less surprising. From the perspectives of adolescents themselves, the observation or experience of CDS symptoms may contribute to insufficient or low-quality sleep, perhaps due to having difficulty “turning off” daydreaming or mind wandering. Alternatively, poor sleep may lead to increased CDS symptoms, such as hypoactivity, lethargy, or mental confusion. Notably, only one prior longitudinal study has examined the directionality of the association between CDS and sleep, finding that insomnia in childhood predicted CDS symptoms in adolescence, but CDS symptoms did not predict future sleep problems [53]. This suggests that persistent sleep difficulties may be risk factors for CDS symptoms, and it will be important for future research to clarify these causal possibilities (see also [8] for experimental findings linking shortened sleep duration to increased CDS symptom severity), as well as test mechanisms linking CDS to more specific aspects of nighttime sleep (e.g., sleep onset latency, sleep quality, night wakings).

In addition, across all three informants (i.e., caregiver, teacher, adolescent), CDS symptoms were uniquely associated with self-reported sleep-related impairment (e.g., daytime sleepiness and related functional impairment). Previous research has evidenced the strong association between CDS and daytime sleepiness [10, 33, 57]. The symptom profile of CDS includes hypoactivity, mental confusion, daydreaming, and so our findings are in line with expectations and previous research linking CDS with sleep-related impairment [40, 65]. It is also important to note that overlapping items were removed prior to conducting analyses in the current study, bolstering confidence in our finding that CDS and sleep-related impairment are distinct but strongly related. The association between teacher-reported CDS symptoms and daytime sleep-related impairment is notable given that these behaviors are temporally aligned and more likely to occur in the school context where teachers are able to observe. In addition, caregiver-reported CDS was significantly associated with caregiver-reported sleep-related impairment. Caregivers may see daytime impairment associated with CDS outside of the school context, such as difficulties in morning routines, afternoon activities, and weekends. In particular, considerable morning difficulty has been identified by caregivers of youth with CDS in previous qualitative and case studies [7, 9].

Despite previous research indicating a significant association between ADHD and sleep difficulties [3, 44], few studies have included CDS symptoms in their analyses. Notably, studies that have included CDS have found CDS symptoms to be a stronger predictor than ADHD-IN of daytime sleepiness [41, 45]. In the current study, ADHD dimensions were frequently not significantly associated with sleep disturbance or sleep-related impairment in the regression models that also included CDS. Specifically, caregiver- and teacher-reported ADHD-IN symptoms were not significantly associated with any of the sleep outcomes, whereas self-reported ADHD-IN was significantly associated with all self-reported sleep variables and caregiver-reported sleep impairment. These findings highlight the importance of obtaining self-report of ADHD symptoms in youth, a practice that is much less common compared to using caregiver- or teacher-report [55]. In addition, there was some evidence of ADHD-HI symptoms being associated with sleep disturbance and sleep-related impairment, but findings were inconsistent across informants and domains examined. These results suggest that the often-noted association between ADHD symptoms and sleep difficulties [47] may be at least in part attributable to co-occurring CDS symptoms. Specifically, youth with ADHD and elevated CDS symptoms may have a distinct profile of sleep-related difficulties that includes delayed sleep onset latency due to mind wandering, whereas other youth with ADHD but low CDS symptoms may have other sleep-related difficulties, a possibility warranting investigation in future research.

Lastly, this study is only the third to examine CDS in relation to circadian preference in adolescents. In contrast to Fredrick et al. [33] and Lunsford-Avery et al. [48], the current study found that self-reported ADHD-IN symptoms, but not CDS symptoms, were significantly associated with circadian preference. Fredrick et al. included adolescents between the ages 12–14, whereas our sample was slightly younger (ages 10–12), though Lunsford-Avery et al. included adolescents spanning a wider age range (ages 13–17). Additional studies are needed to further test the possible link between CDS and circadian preference, with longitudinal studies that simultaneously track pubertal development and other possibly important factors (e.g., school start time) especially needed. Nevertheless, our finding that ADHD-IN is significantly associated with greater eveningness preference is consistent with previous research examining ADHD based on either diagnostic presentation or symptom dimensions [21, 67]. In a large sample of college students, higher ADHD inattentive symptoms were more strongly associated than hyperactive-impulsive symptoms with greater eveningness preference [13]. In the same sample, college students with elevated ADHD combined and predominantly inattentive presentations had higher rates of evening preference compared to those without elevated ADHD symptoms [13]. Among adolescents, ADHD combined presentation has been found to be significantly associated with circadian rhythm sleep disorder, whereas ADHD-IN has been found to be significantly associated with hypersomnia [47]. Additional studies will be needed to further establish the extent to which CDS and/ or ADHD dimensions are associated with circadian function, ideally using longitudinal and multi-method (e.g., dim light melatonin onset) approaches.

### Strengths and Limitations

Strengths of the study include use of multiple informants and psychometrically strong measures in our assessment of CDS, ADHD, depression, and several domains of sleep functioning in a sample of adolescents. Another strength is the inclusion of both ADHD and depressive symptoms, allowing us to find unique associations between CDS and sleep domains, above and beyond symptoms that have been previously found to have strong associations with sleep difficulties. Importantly, we removed sleep-related items from our CDS measures, reducing potential conceptual overlap with our sleep functioning outcomes and strengthening confidence in the specificity of those findings. Limitations include the cross-sectional nature of our data which limits our ability to identify directionality in the association between CDS and sleep functioning, though previous research suggests shortened sleep worsens CDS symptoms [8]. Additional longitudinal and prospective observational studies are needed to inform causality. In addition, our study relied on rating scale measures to assess CDS, ADHD, depression, and sleep functioning. Although we included depressive symptoms in our analyses, we were not able to remove items reflecting low energy or sleep difficulties from the depression scales (as item-level data were unavailable for the BASC which was scored via the publisher’s administration portal), which may have impacted associations with our sleep outcomes. Future research should consider examining these associations using depression measures that omit sleep-related items. Future research should incorporate other measures of sleep functioning, including polysomnography, actigraphy, the multiple sleep latency test, or daily sleep diaries. Further, we used a global measure of sleep disturbance, and it would be beneficial for future research to also examine specific aspects of sleep (e.g., sleep onset, duration, quality, timing) which may evidence differential associations with CDS, ADHD and/or depression.

## Conclusion

The current study extends our current understanding of the association between sleep functioning and CDS. Across informants, CDS was significantly associated with adolescents’ sleep-related impairment, above and beyond symptoms of ADHD and depression. In addition, caregiver- and teacher-reported CDS was uniquely associated with caregiver-reported sleep-related impairment, and adolescents’ self-reported CDS was uniquely associated with sleep disturbance. This study further demonstrates the distinct nature of CDS and ADHD in relation to sleep functioning, highlighting the need to include CDS symptoms in future research on ADHD and sleep functioning. Future longitudinal research is needed to further delineate the directionality of these associations, particularly across adolescence.

## Supplementary Material

Supplemental Material

**Supplementary Information** The online version contains supplementary material available at https://doi.org/10.1007/s10578-026-01990-z.

## Figures and Tables

**Table 1:** Sample Characteristics (*N* = 341)

Adolescent Characteristics		Caregiver/Family Characteristics	
	M ± SD		*n* (%)
Age	10.90 ± 0.80	Relationship to Child	
		Biological Mother	293 (85.9)
	*n* (%)	Biological Father	26 (7.6)
Sex		Stepmother	2 (0.6)
Female	178 (52.2)	Adoptive Mother	14 (4.1)
Male	163 (47.8)	Adoptive Father	1 (0.3)
		Foster Mother	1 (0.3)
Race		Grandmother	3 (0.9)
American Indian/Alaskan	1 (0.3)	Grandfather	1 (0.3)
Asian	8 (2.3)		
Black	72 (21.1)	Household Income^b^	
Multiracial	48 (14.1)	Under $20,000	15 (4.5)
White	212 (62.2)	20,001–40,000	37 (11.1)
		40,001–60,000	34 (10.2)
Hispanic/Latinx	32 (9.4)	60,001–80,000	38 (11.4)
		80,001–100,000	34 (10.2)
Psychiatric diagnoses^a^		100,001–120,000	45 (13.5)
ADHD - CR	166 (48.7)	Over $120,000	130 (39.0)
Any externalizing (ODD)^a^ - CR	24 (7.0)		
Any anxiety - CR	52 (15.2)	Highest Primary Caregiver Education^c^	
Any anxiety - SR	39 (11.4)	High school degree or less	25 (7.4)
Any depression - CR	5 (1.5)	Partial college/vocational	51 (15.1)
Any depression - SR	8 (2.3)	College graduate	141 (41.7)
		Graduate/professional degree	121 (35.8)
Medication Use			
ADHD	83 (24.3)		
Other psychiatric	24 (7.0)		

ADHD = attention-deficit/hyperactivity disorder. ODD = oppositional defiant disorder. Anxiety disorders=presence of generalized anxiety disorder, social phobia, panic disorder, and/or posttraumatic stress disorder (PTSD). Any depression=presence of major depression or dysthymia CR=caregiver-report. SR=adolescent self-report

a Current psychiatric diagnoses were assessed using the Kiddie Schedule for Affective Disorders and Schizophrenia for School-Age Children (K-SADS). No participants met criteria for conduct disorder

b Eight caregivers did not provide family income

c Three caregivers did not provide education

**Table 2:** Bivariate Correlations and Descriptive Statistics of Primary Study Variables

Variable	1	2	3	4	5	6	7	8	9	10	11	12	13	14	15	16	17
1. CR CDS	--																
2. CR IN	0.71***	--															
3. CR HI	0.47***	0.69***	--														
4. CR DEP	0.45***	0.35***	0.25***	--													
5. TR CDS	0.36***	0.33***	0.22***	0.15*	--												
6. TR IN	0.25***	0.41***	0.36***	0.12*	0.70***	--											
7. TR HI	0.10	0.23***	0.34***	0.03	0.22***	0.60***	--										
8. TR DEP	0.18**	0.13*	0.11	0.22***	0.43***	0.34***	0.17**	--									
9. SR CDS	0.36***	0.36***	0.32***	0.23***	0.25***	0.22***	0.16**	0.21***	--								
10. SR IN	0.37***	0.49***	0.38***	0.19***	0.25***	0.33***	0.29***	0.15*	0.69***	--							
11. SR HI	0.21***	0.35***	0.44***	0.16***	0.18**	0.36***	0.40***	0.14*	0.58***	0.74***	--						
12. SR DEP	0.27***	0.27***	0.19***	0.42***	0.14*	0.20***	0.18**	0.20***	0.41***	0.48***	0.49***	--					
13. CR Sleep Disturbance	0.35***	0.33***	0.34***	0.32***	0.09	0.10	0.11	0.12*	0.29***	0.28***	0.22***	0.19***	--				
14. CR Sleep Impairment	0.41***	0.33***	0.21***	0.40***	0.22***	0.15*	0.10	0.26***	0.26***	0.25***	0.13*	0.19***	0.64***	--			
15. SR Sleep Disturbance	0.19**	0.19***	0.17**	0.20***	0.15**	0.21***	0.23***	0.15*	0.45***	0.47***	0.44***	0.52***	0.29***	0.24***	--		
16. SR Sleep Impairment	0.28***	0.24***	0.18**	0.30***	0.24***	0.19**	0.10	0.24***	0.44***	0.45***	0.40***	0.51***	0.19***	0.28***	0.70***	--	
17. Circadian Preference	− 0.03	− 0.03	− 0.02	− 0.13*	− 0.06	− 0.11	− 0.13*	− 0.09	− 0.27***	− 0.32***	− 0.23***	− 0.18***	− 0.07	− 0.22***	− 0.35***	− 0.43***	--
*Mean*	1.07	1.98	1.31	0.34	1.12	1.40	0.85	0.33	1.06	9.82	9.43	4.45	1.89	1.62	2.01	2.24	27.95
*SD*	1.01	1.47	1.28	0.53	1.03	1.34	1.16	0.61	0.54	5.51	5.12	4.29	0.78	0.64	0.77	0.69	5.36
*Skewness*	0.89	0.22	0.82	2.33	1.07	0.92	1.51	3.34	0.41	0.14	0.44	1.63	0.76	0.92	1.04	0.86	− 0.24
*Kurtosis*	− 0.08	−1.23	− 0.40	6.44	0.53	− 0.23	1.20	13.86	− 0.07	− 0.86	− 0.13	2.88	− 0.34	0.02	0.57	0.15	− 0.05

Note. *N* = 341. IN=attention-deficit/hyperactivity disorder inattention. HI=attention-deficit/hyperactivity disorder hyperactivity-impulsivity. DEP = depression. CDS=cognitive disengagement syndrome. CR=caregiver-report. SR=self-report. TR=teacher-report

* *p*<.05.

** *p*<.01.

*** *p* < .001

**Table 3:** Multivariate Regression Analyses Examining Depression, ADHD Dimensions, and CDS Symptoms in Relation to Sleep and Circadian Preference Outcomes

Informant (IVs)	Caregiver-Reported Outcomes				Adolescent Self-Reported Outcomes					
	Sleep Disturbance		Sleep-Related Impairment		Sleep Disturbance		Sleep-Related Impairment		Circadian Preference	
	Coefficient	SE	Coefficient	SE	Coefficient	SE	Coefficient	SE	Coefficient	SE
CR Symptoms	*R*^2^ = 0.20, *p*<.001		*R*^2^ = 0.25, *p*<.001		*R*^2^ = 0.10, *p*=.002		*R*^2^ = 0.17, *p*<.001		*R*^2^ = 0.04, *p*=.053	
DEP	0.20***	0.06	0.28***	0.05	0.10	0.06	0.17**	0.06	− 0.13*	0.06
CDS	0.12	0.08	0.19**	0.07	0.09	0.08	0.18*	0.08	0.05	0.08
ADHD-IN	0.05	0.09	0.13	0.09	0.02	0.09	− 0.01	0.09	− 0.03	0.10
ADHD-HI	0.20**	0.07	− 0.04	0.07	− 0.01	0.08	− 0.07	0.07	0.03	0.08
TR Symptoms	*R*^2^ = 0.08, *p*=.007		*R*^2^ = 0.12, *p*=.001		*R*^2^ = 0.12, *p*=.001		*R*^2^ = 0.16, *p*<.001		*R*^2^ = 0.05, *p*=.045	
DEP	0.05	0.07	0.18**	0.07	0.07	0.07	0.16*	0.06	− 0.04	0.07
CDS	0.03	0.09	0.19*	0.09	0.07	0.09	0.19*	0.09	− 0.01	0.09
ADHD-IN	− 0.03	0.11	− 0.13	0.11	0.01	0.10	− 0.09	0.10	− 0.05	0.11
ADHD-HI	0.08	0.08	0.09	0.08	0.17*	0.08	0.02	0.07	− 0.09	0.08
SR Symptoms	*R*^2^ = 0.13, *p*<.001		*R*^2^ = 0.13, *p*<.001		*R*^2^ = 0.35, *p*<.001		*R*^2^ = 0.35, *p*<.001		*R*^2^ = 0.12, *p*<.001	
DEP	0.06	0.06	0.12	0.06	0.36***	0.05	0.35***	0.05	− 0.05	0.06
CDS	0.17*	0.07	0.14	0.07	0.17**	0.06	0.19**	0.06	− 0.07	0.07
ADHD-IN	0.13	0.09	0.22*	0.09	0.12	0.08	0.14	0.08	− 0.28**	0.09
ADHD-HI	− 0.05	0.08	− 0.22**	0.08	0.07	0.07	− 0.03	0.07	0.04	0.08

*N* = 341. All analyses include sex, race, family income, and medication status as covariates. ADHD-HI=attention-deficit/hyperactivity disorder hyperactivity-impulsivity. ADHD-IN=attention-deficit/hyperactivity disorder inattention. CDS=cognitive disengagement syndrome. DEP = depression. IVs = independent variables. CR=caregiver-report. SR=self-report. TR=teacher-report

* *p*<.05.

** *p*<.01.

*** *p* < .001

## Data Availability

Data preparation and descriptive statistics were done in SPSS 28. Primary analyses were conducted in Mplus. Data and code are available from the corresponding author upon reasonable request and execution of a data use agreement.
