# Supplementary material for: Multi-informant Examination of Cognitive Disengagement Syndrome in Relation to Sleep and Circadian Preference in Early Adolescents
Source: Child Psychiatry Hum Dev. Author manuscript; Available in PMC 2026 Sep 27. (PMC13616222; doi:10.1007/s10578-026-01990-z)
Supplement: Supplementary file 1 [file NIHMS2207112-supplement-Supplemental_Material.docx]

SUPPLEMENTAL MATERIALS

Table S1

*Correlations of Covariate Variables with Primary Study Variables*

| Variable | 1 | 2 | 3 | 4 |
| --- | --- | --- | --- | --- |
| 1. Medication | -- |  |  |  |
| 2. Sex | -.15** | -- |  |  |
| 3. Race | .14** | -.02 | -- |  |
| 4. Income | .07 | -.07 | .26*** | -- |
| 5. CR CDS | .24*** | -.09 | .10 | -.18** |
| 6. CR IN | .46*** | -.19*** | .05 | -.08 |
| 7. CR HI | .47*** | -.18** | .00 | -.15** |
| 8. CR DEP | .28*** | .01 | .03 | -.07 |
| 9. TR CDS | .18** | -.15* | .01 | -.14* |
| 10. TR IN | .24*** | -.28*** | -.10 | -.17** |
| 11. TR HI | .20** | -.20** | -.11 | -.16** |
| 12. TR DEP | .11 | .08 | -.09 | -.24*** |
| 13. SR CDS | .26*** | .04 | -.05 | -.17** |
| 14. SR IN | .33*** | -.04 | -.07 | -.12* |
| 15. SR HI | .34*** | -.04 | -.12* | -.15** |
| 16. SR DEP | .31*** | .01 | -.03 | .00 |
| 17. CR Sleep Disturbance | .17** | -.02 | .03 | -.17** |
| 18. CR Sleep Impairment | .12* | -.02 | .00 | -.19** |
| 19. SR Sleep Disturbance | .22*** | .00 | -.08 | -.08 |
| 20. SR Sleep Impairment | .27*** | -.05 | -.10 | -.11* |
| 21. Circadian Preference | -.05 | -.07 | .10 | .11* |
| *Mean* | -- | -- | -- | 5.08 |
| *SD* | -- | -- | -- | 1.99 |
| *Skewness* | -- | -- | -- | -.59 |
| *Kurtosis* | -- | -- | -- | -1.06 |

*Note*. *N* = 341. For medication use, 0 = not currently taking any prescribed psychiatric medication, 1 = currently taking a prescribed psychiatric medication. For sex, 0 = male, 1 = female. For race, 0 = person of color, 1 = White. For income, an ordinal indicator was used with 1 = under $20,000, 7 = over $120,000. HI=attention-deficit/hyperactivity disorder hyperactivity-

impulsivity. IN=attention-deficit/hyperactivity disorder inattention. CDS=cognitive disengagement syndrome. DEP = depression. CR=caregiver-report. SR=self-report. TR=teacher-report. **p*<.05. ***p*<.01. ****p* < .001.
